# Learning from previous lockdown measures and minimising harmful biopsychosocial consequences as they end: A systematic review

**DOI:** 10.7189/jogh.11.05008

**Published:** 2021-05-22

**Authors:** Paula A Muehlschlegel, Edward AJ Parkinson, Randell YL Chan, Madelynne A Arden, Christopher J Armitage

**Affiliations:** 1 Royal Free London NHS Foundation Trust, London, UK; 2 York Teaching Hospital NHS Foundation Trust, York, UK; 3 The University of Manchester, Manchester, UK; 4 Centre for Behavioural Science and Applied Psychology, Sheffield Hallam University, Sheffield, UK; 5 Manchester Centre for Health Psychology, The University of Manchester, Manchester, UK; 6 Manchester Academic Health Science Centre, Manchester University NHS Foundation Trust, Manchester, UK

## Abstract

**Background:**

Infectious outbreaks, most recently coronavirus disease 2019 (COVID-19), have required pervasive public health strategies, termed lockdown measures, including quarantine, social distancing, and closure of workplaces and educational establishments. Although evidence analysing immediate effects is expanding, repercussions following lockdown measures remain poorly understood. This systematic review aims to analyse biopsychosocial consequences after lockdown measures end according to short, medium, and long-term impacts.

**Methods:**

PubMed, Ovid MEDLINE, Embase, PsycInfo, Web of Science, and Scopus databases were searched from inception to January 12, 2021. Reference lists were manually reviewed. Eligible studies analysed biopsychosocial functioning after lockdown measures secondary to recent infectious outbreaks ended. Lockdown measures were defined as quarantine, isolation, workplace or educational closures, social or physical distancing, and national or local closure of public institutions deemed non-essential. Studies exclusively researching outcomes during lockdown measures, examined infectious participants, or analysed lockdown measures not pertaining to an infectious outbreak were excluded. Two independent reviewers extracted data and assessed bias with a third resolving discrepancies. Data was extracted from published reports with further information requested from authors where necessary. The mixed methods appraisal tool assessed study quality, languages were restricted to English, German, Italian, and French and narrative synthesis was applied.

**Results:**

Of 5149 identified studies, 40 were eligible for inclusion. Psychological distress, economic repercussions, social, biological, and behavioural ramifications were observed. Short to medium-term effects comprised reactions relating to early trauma processing whereas medium to long-term repercussions manifested in maladaptive behaviours and mental health deterioration. Increased alcohol intake, stigmatisation, and economic effects were also identified consequences. High-risk groups included health care workers, children, elderly, inpatients, those with pre-existing psychiatric diagnoses, and socially isolated individuals.

**Conclusions:**

Supporting vulnerable groups and offering education, workplace modifications, financial, and social assistance may mitigate negative repercussions. Establishing a rapid and comprehensive evidence base appraising the efficacy of such interventions and identifying areas for development is essential. This review was limited by study heterogeneity and lack of randomisation in available literature. Given the unprecedented nature and progression of COVID-19, the relevance of previous outcomes remains uncertain.

**Protocol registration:**

PROSPERO registration CRD42020181134

The coronavirus disease 2019 (COVID-19) pandemic has had exceptional global impacts requiring stringent public health measures known as “lockdowns” [1-3]. Lockdown measures, encompassing publicly enforced home, hospital or workplace quarantine, physical distancing, isolation, and closure of public buildings and educational institutions, have been used to contain infectious diseases but have not been implemented at scale since 1920 [2,4-7]. Infectious outbreaks, including severe acute respiratory syndrome (SARS), Middle East respiratory syndrome (MERS), Ebola, swine flu (H1N1), and COVID-19, have reaffirmed such measures as effective in mitigating the destructive potential of infectious diseases, however, may precipitate challenges following their removal [8]. Literature on the immediate impacts of lockdowns and the effects of the COVID-19 pandemic on physical well-being, mental health, and economic climate is rapidly growing [4,9-11]. Brooks *et al* have reviewed literature regarding psychological impacts during quarantine, describing considerable mental health burden including anxiety, depression, and post-traumatic stress disorder (PTSD) symptoms [4]. Closure of workplaces, educational institutions, and leisure activities have significantly disrupted personal and professional pursuits with ensuing lack of social normalcy, loss of academic achievements, and decreased physical activity evoking predictions of persistent ramifications on social functioning and physical health [5,12,13]. Delays in investigations, diagnoses, and treatment have additionally generated concerns regarding indirect morbidity and mortality [14]. As we move beyond the acute phase of the COVID-19 pandemic, the question arises as to what happens when lockdown measures end, for how long impacts persist, and whether certain groups are disproportionately affected. Previous research suggests persistent biopsychosocial consequences up to 3 years after lockdown measures end including effects on mental health with symptoms of anxiety disorders, depression, and PTSD described [15-32]. Social and behavioural repercussions such as discrimination, changes in dietary behaviours, economic effects, and biological impacts including alterations to sleep, biochemical parameters, and weight have additionally been determined [29,33-42]. Certain groups including health care workers (HCWs) and those with pre-existing psychiatric conditions have been identified as high-risk [15,17,27,31,32,35,41,43-45].

The aim of this systematic review is, for the first time, to comprehensively evaluate biopsychosocial consequences after lockdown measures are lifted and identify strategies to successfully negotiate the transition out of COVID-19 related lockdowns. We hypothesise that lockdown measures can have extensive biopsychosocial consequences, may disproportionately affect certain groups, and could vary according to the time elapsed after their end. To identify what happens following lockdown, and if repercussions vary according to the time passed, this review will categorise outcomes according to short-term, occurring within one month, medium-term from one to six months and long-term after six months. Outcomes will additionally be stratified according to disease outbreak and impacts on families and children are separately described.

## METHODS

### Search strategy and selection criteria

The protocol was designed according to the Preferred Reporting Items for Systematic Reviews and Meta-Analyses statement and registered on PROSPERO, CRD42020181134 [46]. Two reviewers independently conducted systematic online literature searches from database inception to January 12, 2021, utilising the population, intervention, comparison, outcome framework on PubMed, Ovid MEDLINE, Embase, PsycInfo, Web of Science, and Scopus databases. Search terms are described in Table S1 in the **Online Supplementary Document**. Studies were filtered to clinical trials, human populations, and languages restricted to English, German, Italian, or French. In the present review, lockdown measures encompassed home, hospital, workplace, dormitory, or camp quarantine, knowing somebody who had been quarantined, social and physical distancing, and national or local closure of public institutions deemed non-essential. Reviewed studies investigated biopsychosocial outcomes after lockdown measures ended including effects on mental health, sleep, weight, biochemical markers, social connectivity, and stigmatisation. The definition of stigmatisation varied from subjective discrimination to objective social ostracization. Lockdown measures must have been implemented following an infectious outbreak, namely SARS, COVID-19, MERS, Ebola, or H1N1. Descriptive studies were included in the absence of a comparator to lockdown measures where these were lacking. Reference lists of identified studies were manually examined. Studies were excluded if they assessed biopsychosocial outcomes exclusively during lockdown measures, involved non-human participants, were review articles, or included populations that exhibited symptoms suggestive of the associated disease.

### Data extraction and synthesis

Two reviewers independently identified studies and extracted data to Microsoft Excel (Microsoft Inc, Seattle WA, USA). Discrepancies were discussed and resolved with a third reviewer when a consensus was not established. Authors were contacted for further information where necessary. Significant heterogeneities in study design, lockdown measures, outcomes, and measurement tools prevented *I^2^* calculation and quantitative meta-analysis, therefore narrative synthesis was applied. The ENTREQ statement was utilised [47]. Studies were pooled according to time elapsed after lockdown measures ended; namely, short-term ensuing within one month, medium-term arising between one to six months and long-term defined at over one year, disease cause, and outcomes. Timelines for stratification were chosen in agreement with the diagnostic and statistical manual of mental disorder criteria for conditions including trauma and stress related disorders (symptom onset within one month), PTSD (duration of disturbance for over one month), and generalised anxiety disorder (symptom duration for at least six months) [48]. Symptoms were clustered using international classifications of mental disorders where possible. As few studies examined families and children (defined as under 18 years old), these were described separately. Recommendations for mitigating biopsychosocial effects were made following analysis of risk factors and ordinary interventions.

### Bias assessment

The Mixed Methods Appraisal Tool (MMAT) was used by two independent reviewers to determine study quality with a third resolving discrepancies [49]. Quality was calculated using five domains scoring one point per domain achieved to a total of 5, with 5 equating to the highest study quality and 0 the lowest. Study designs were defined as qualitative, quantitative randomised controlled trial, quantitative non-randomised, quantitative descriptive, or mixed methods. Investigated domains for potential bias differed for each study design; however, all included analysis of participant selection, data collection, interpretation, and data reporting. No variation was made to study interpretation based on MMAT scores of 3 or more although study findings scoring 2 were assessed more critically. Studies scoring 0 or 1 were excluded.

### Study registration

PROSPERO registration CRD42020181134.

### Funding

Funding was not sourced for this study.

### Patient and public involvement

This systematic review contained no direct patient and public involvement.

## RESULTS

### Study characteristics

**Figure 1:**
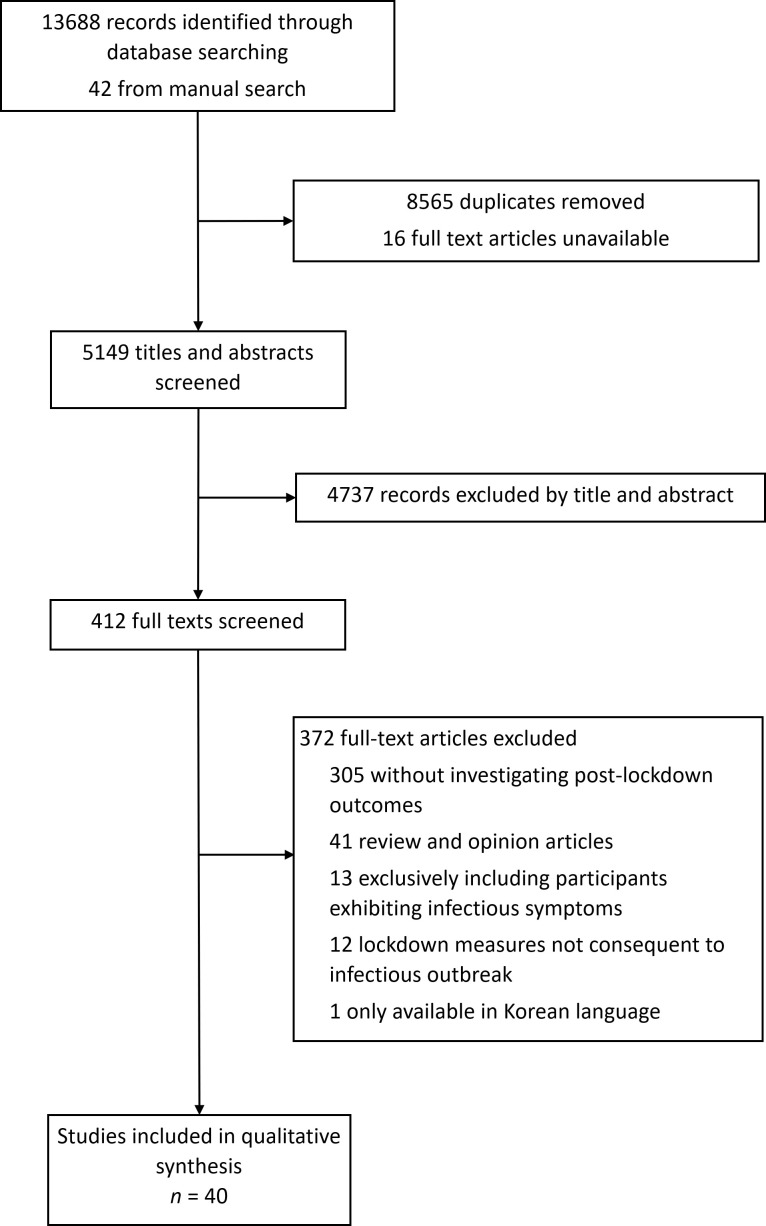
Study selection.

40 studies met inclusion criteria (**Figure 1**). Study designs comprised cross-sectional (n = 20), longitudinal (n = 10), qualitative (n = 4), cohort (n = 2), mixed methods (n = 2), case report (n = 1), and case-control (n = 1) (**Table 1**). Data was collected through questionnaire surveys (n = 30), focus groups or interviews (n = 5), biochemical testing (n = 3), audit of service utilisation (n = 1), and case note review (n = 1). Analysed studies assessed outcomes following lockdown measures related to infectious outbreaks, namely SARS (n = 17), COVID-19 (n = 15), MERS (n = 6), Ebola (n = 1), and H1N1 (n = 1). Implemented lockdown measures varied according to disease, country, and participant professional backgrounds, however, included one or a combination of home quarantine (n = 17), national lockdown with social distancing measures (n = 9), workplace quarantine (n = 8), hospital quarantine (n = 7), citywide lockdown (n = 4), workplace closure or suspension of elective surgical procedures (n = 2), dormitory quarantine (n = 1), contact with quarantined individuals (n = 1), and camp isolation (n = 1). Studies sampled diverse age groups (6 to over 65 years), various countries, and differing populations. Sample sizes ranged from 10 to 6231 participants. One study was excluded based on language. No studies scored 0 or 1 using the MMAT. Further studies scored 2 (n = 5), 3 (n = 19), 4 (n = 9), and 5 (n = 7). Where potential biases were evident, these were due to lack of baseline data, confounding factors, variable implementation of lockdown measures, and use of self-reporting as a measurement tool (Appendix S2 in the **Online Supplementary Document**).

**Table 1:** Study characteristics

Author	Country	Design	Participants	Lockdown measure	Lockdown measure duration	Post-lockdown measure assessment time point*	Disease	Primary outcomes	Measurement tools	Quality rating†
Bai et al (2004) [15]	Taiwan	Cross-sectional	338 health care workers	Quarantine (type unclear)	9 days	Up to 10 days	SARS	Acute stress disorder	Study-specific questionnaire adapted from DSM-IV	3
Cava et al (2005) [39]	Canada	Qualitative	21 Toronto residents	Home quarantine	9 days (mean length)	Up to several months	SARS	Stigma, behavioural changes and psychological well-being	Interview	5
Chandola et al (2020) [50]	UK	Longitudinal	Between 13754 and 17761 adults in the general population	UK national lockdown	3-4 months	3-4 weeks	COVID-19	Common mental disorders	General Health Questionnaire-12	4
Chen et al (2007) [44]	Taiwan	Cohort study	172 hospital staff; 90 health care workers caring for SARS patients and 82 administrative staff	Home quarantine and working non-SARS shifts	28 days (14 days quarantine, 14 days non-SARS shifts)	2 weeks	SARS	Physical functioning, role physical, bodily pain, vitality, role emotional, social functioning, general health and mental health	Medical Outcomes Study 36-Item Short Form Survey	3
Cho et al (2020) [16]	South Korea	Cross-sectional	67 haemodialysis patients	Home or hospital quarantine in a single room or cohort ward	14.8 days (mean length)	1 year	MERS	PTSD	Impact of Event Scale-Revised Korean version	3
Chong et al (2004) [45]	Taiwan	Cross-sectional	1257 health care workers, 79 of whom had been quarantined	Dormitory quarantine	14 days	Up to 7 weeks	SARS	PTSD and mental health	Impact of Event Scale and Chinese Health Questionnaire	2
Daly & Robinson (2020) [22]	USA	Longitudinal	7319 nationally representative adults	USA national and state-level lockdown	Variable by state	1-3 months	COVID-19	Psychological distress	Patient Health Questionnaire-4	5
DiGiovanni et al (2004) [40]	Canada	Qualitative	1509 Toronto residents, 6 focus groups with 9- 13 persons each	Home quarantine	10 days	4 months	SARS	Stigma, behavioural changes and psychological well-being	Focus group	4
Duy et al (2020) [51]	Vietnam	Cross-sectional	61 health care workers	Hospital quarantine	23 days	6-9 days	COVID-19	Stigma, depression, anxiety and stress	Study-specific questionnaire adapted from HIV Stigma Scale and Depression, Anxiety and Stress Scale-21	4
Grigoletto et al (2020) [52]	Italy	Longitudinal	338 adolescent and young adult emergency department attendances	Italy national lockdown	2-3 months	Up to 3 weeks	COVID-19	Severe alcohol intoxications	Blood alcohol content	5
Grover et al (2020) [21]	India	Cross-sectional	144 ophthalmologists	India national lockdown and postponement of elective surgeries	6 weeks	Up to 2 weeks	COVID-19	Depression, anxiety and stress	Depression, Anxiety and Stress Scale-21	3
Hawryluck et al (2004) [26]	Canada	Cross-sectional	129 Toronto residents, 88 of whom were health care workers	Home or workplace quarantine	10 days (median length)	Median 36 days	SARS	PTSD and depression	Impact of Event Scale-Revised and Center for Epidemiological Studies-Depression	3
Jalloh et al (2018) [25]	Sierra Leone	Cross-sectional	3564 members of the general population, 1165 of whom had known somebody quarantined	Quarantine (type unclear)	21 days	Up to 14 months	Ebola	PTSD, anxiety and depression	Impact of Event Scale-Revised and Patient Health Questionnaire-4	3
Jeong et al (2016) [27]	South Korea	Longitudinal	1656 South Korean residents	Home, workplace or hospital quarantine	14 days	4-6 months	MERS	Anxiety and anger	General Anxiety Disorder-7 and State-Trait Anger Expression Inventory-2	4
Kim et al (2019) [33]	South Korea	Longitudinal	83 haemodialysis patients and 12 health care workers	Home or hospital quarantine	17 days	1 & 3 months	MERS	Physical and emotional stress and haemodialysis efficacy	Multiple plasma biochemical markers	3
Ko et al (2006) [28]	Taiwan	Cross-sectional	1499 individuals, 398 of whom exhibited isolated behaviour	Quarantine (type unclear)	Length unclear	1 months	SARS	Depression	Taiwanese Depression Questionnaire	2
Koller et al (2006) [53]	Canada	Qualitative	23; 10 parents, 5 children and 8 health care workers	Hospital quarantine	Length unclear	Up to several months	SARS	Behavioural changes and psychological well-being	Interview	5
Lee et al (2005) [36]	Hong Kong	Mixed methods	903 Hong Kong residents	Isolation camp quarantine	3-4 months	1-5 months	SARS	Stigma	Study-specific questionnaire	3
Lee et al (2018) [35]	South Korea	Longitudinal	359 health care workers working during hospital shutdown and 77 health care workers 1 months following shutdown	Hospital lockdown	24 days	1 months	MERS	PTSD	Impact of Event Scale-Revised Korean version	3
Lei et al (2020) [30]	China	Cross-sectional	1593 individuals in Southwestern China, 420 of whom had been affected by quarantine	Quarantine (type unclear)	Length unclear	Up to 3 weeks	COVID-19	Anxiety and depression	Self-rating Anxiety Scale and Self-rating Depression Scale	4
Li et al (2020) [23]	China	Longitudinal	173 university students	China national, provincial and citywide lockdowns	2-3 months	Up to 3 months	COVID-19	Depression, anxiety, stress and novelty seeking	Depression, Anxiety and Stress Scale-21 and Langer Mindfulness Scale novelty seeking subscale	3
Liu et al (2012) [31]	China	Cross-sectional	549 health care workers, 103 of whom had been quarantined	Home or workplace quarantine	Length unclear	3 years	SARS	Depression	Center for Epidemiological Studies-Depression	3
Lu et al (2020) [24]	China	Cross-sectional	1417 Wuhan residents; 387 health care workers and 1035 members of the general population	China national, provincial and citywide lockdowns	2-3 months	2 months	COVID-19	PTSD, anxiety and depression	PTSD Checklist-Civilian version, General Anxiety Disorder-7 and Patient Health Questionnaire-9	4
Marjanovic (2007) [37]	Canada	Cross-sectional	333 nurses	Home or workplace quarantine	Length unclear	Up to 7 months	SARS	Emotional exhaustion, anger and avoidance behaviour	Maslach Burnout Inventory-General Survey, State-Trait Anger Expression Inventory-2 and study-specific questionnaire	4
Mihashi et al (2009) [54]	China	Cross-sectional	187; printing company workers, university faculty staff and students	Citywide lockdown	Length unclear	7-8 months	SARS	Psychological disorders	General Health Questionnaire-30	3
Park et al (2020) [38]	South Korea	Cohort study	116 haemodialysis patients	Home or hospital quarantine in a single room or cohort ward	15 days (mean length)	3-6 months	MERS	Clinical parameters and haemodialysis efficacy	Blood pressure and multiple plasma biochemical markers	3
Ping et al (2008) [43]	China	Cross-sectional	549 health care workers, 103 of whom had been quarantined	Home or workplace quarantine	Length unclear	3 years	SARS	Alcohol abuse and dependence	National Household Survey on Drug Abuse	3
Ping et al (2009) [32]	China	Cross-sectional	549 health care workers, 103 of whom had been quarantined	Home or workplace quarantine	Length unclear	3 years	SARS	PTSD	Impact of Event Scale-Revised	3
Probst et al (2020) [20]	Austria	Longitudinal	445 adults in the general population	Austria national lockdown	2-3 months	1-3 weeks	COVID-19	Depression	Patient Health Questionnaire-9	3
Reynolds et al (2007) [17]	Canada	Cross-sectional	1057; 269 health care workers, 291 patients, 327 visitors, 139 other	Home or workplace quarantine	8.3 days (median length)	1-4 months	SARS	PTSD, stigma, behavioural changes and psychological well-being	Impact of Event Scale-Revised and study-specific questionnaire	5
Ritish et al (2020) [29]	India	Cross-sectional	1602 international air passengers	Home or institutional quarantine	14 days	Up to 1 week	COVID-19	Anxiety, depression, suicidal ideation and sleep disturbance	Interview	3
Robertson et al (2004) [41]	Canada	Qualitative	10 health care workers	Home or workplace quarantine	10 days	Up to 4 months	SARS	Stigma, behavioural changes and psychological well-being	Interview	2
Sprang & Silman (2013) [18]	USA & Canada	Mixed methods	398 parents	Home quarantine and social distancing	Length unclear	1-6 months	H1N1 & SARS	PTSD	PTSD Checklist-Civilian version and PTSD-Reaction Index	2
Tan et al (2020) [19]	China	Cross-sectional	673 workforce members	Workplace closure	14 days	Up to 1 month	COVID-19	PTSD, depression, anxiety, stress, insomnia and psychological well-being	Impact of Event Scale-Revised, Depression, Anxiety and Stress Scale-21, Insomnia Severity Index and study-specific questionnaires	4
Wang et al (2020) [55]	China	Cross-sectional	1210 individuals in cities across China, 26 of whom had been recently quarantined	Home quarantine	Length unclear	Up to 2 weeks	COVID-19	PTSD, depression, anxiety and stress	Impact of Event Scale-Revised and Depression, Anxiety and Stress Scale-21	3
Yip et al (2010)[56]	Hong Kong	Case-control	66 elderly suicides; 22 SARS-related, 44 randomly selected controls	Social distancing	Length unclear	Up to 5 months	SARS	Isolation experience, social contact and psychological well-being	Case-note review	5
Yoon et al (2016)[57]	South Korea	Case report	6231 Gyeonggi residents	Quarantine (type unclear)	Length unclear	Up to several months	MERS	Mental health service utilisation	Audit	5
Zarah et al (2020) [42]	USA	Cross-sectional	3133 American adults	USA national and state-level lockdown	Variable by state	4 months	COVID-19	Dietary habits	Study-specific questionnaire	4
Zhang et al (2020) [34]	China	Longitudinal	1994 Chinese adults	China national, provincial and citywide lockdowns	2-3 months	4 months	COVID-19	Dietary habits	Study-specific questionnaire	3
Zhou et al (2020) [58]	China	Longitudinal	279 Wuhan residents	Citywide lockdown	2-3 months	Up to 6 weeks	COVID-19	Depression, psychological need satisfaction, and loneliness	Center for Epidemiological Studies-Depression, Need Satisfaction Scale and Revised Loneliness Scale	2

SARS – severe acute respiratory syndrome, COVID-19 – coronavirus disease 2019, MERS – Middle East respiratory syndrome, H1N1 – influenza A virus subtype H1N1, PTSD – post-traumatic stress disorder, DSM – Diagnostic and Statistical Manual of Mental Disorders

*Time points calculated from start of infectious outbreak to data collection period if lockdown measure duration unclear.

†Quality score ranged from meeting none of five criteria (0) to meeting all criteria (5).

### Short-term consequences of lockdown measures (≤1 month)

**Figure 2:**
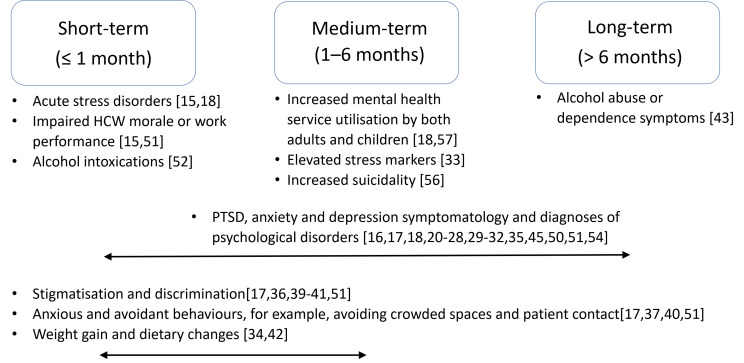
Biopsychosocial consequences identified after lockdown measures end from 40 included studies. HCW – health care worker, PTSD – post-traumatic stress disorder.

13 studies reported short-term consequences of lockdown with psychiatric symptoms, sleep disturbance, economic aftereffects, behavioural changes, and social repercussions described (**Figure 2**) [15,19-21,28-30,35,44,50-52,55].

#### Severe acute respiratory syndrome

Five studies identified short-term repercussions following SARS outbreaks [15,28,36,39,44]. Bai *et al* found that 17% (7 of 41) of Taiwanese HCWs suffered from an acute stress disorder up to ten days following quarantine compared with 4% (10 of 297) of those not quarantined [15]. Moreover, 20% of quarantined HCWs were reluctant to work compared to 5% not experiencing these measures and were more likely to consider resigning or observed a significant deterioration in their work performance [15]. In contrast, Chen *et al* described improved physical, social, and emotional well-being in HCWs after two weeks of self-quarantine and a further two weeks of off-duty shifts in comparison to those surveyed immediately after on-duty shifts treating SARS patients [44]. However, outcomes remained significantly impaired compared to controls lacking patient contact, particularly for vitality and mental health scores [44]. Ko *et al* associated higher depression symptomatology with being isolated, having poorer family or neighbourhood relationships, and negative economic effects following quarantine [28].

Social repercussions, including stigmatisation, discrimination, behavioural, and emotional reactions were short-term after effects [36,39]. Stigma or discrimination, including interpersonal and professional marginalisation, were experienced by 48∙7% (440 of 903) of individuals returning to work in Hong Kong following quarantine [36]. Canadian participants also reported stigmatisation from colleagues and detailed behavioural changes, including vigorous handwashing and avoidance of crowds [39]. Emotional reactions described by Toronto residents following quarantine comprised initial relief, however, participants expressed unease regarding the effect these measures may have on socially isolated and financially insecure populations [39].

#### Middle East respiratory syndrome

PTSD symptoms in HCWs were investigated by Lee *et al* after 24 days of hospital shutdown with 51∙5% (183 of 359) initially meeting diagnostic criteria [35]. One month later, 40∙3% (31 of 77) of survey respondents originally fulfilling criteria remained eligible for PTSD diagnoses [35]. Quarantine itself was not correlated with overall diagnostic scores, however, post-hoc analyses associated higher sleep and numbness sub-scores [35].

#### Coronavirus disease 2019

Psychiatric symptoms, stress, stigmatisation, sleep disturbance, and increased emergency department attendances with alcohol intoxication were identified following COVID-19 lockdown measures [19-21,29,30,50-52,55].

Eight studies investigated mental health repercussions [19-21,29,30,50,51,55]. PTSD (10∙8%, 73 of 673), depression (3∙7%, 25 of 673), and anxiety (3∙8%, 26 of 673) symptoms were identified in returning workforces, with symptom prevalence similar to that of the general population at the start of measures [19]. Strict personal and workplace hygiene and attention to employees physical and psychiatric well-being may have mitigated psychological morbidity [19]. Acute symptoms of anxiety and depression were identified in six studies [20,21,29,30,50,51]. Lei *et al* described significantly increased symptoms of anxiety (12.9%, 54 of 420), and depression (22.4%, 94 of 420), in those quarantined, compared to 6.7% (79 of 1173) and 11.9% (140 of 1173) in unaffected individuals respectively [30]. Identified risk factors for psychiatric symptoms included stress, loneliness, being female, worse self-perceived health, poor physical health, lower educational levels, lower household income, and those experiencing economic fallout [19-21,30,50]. In contrast, Wang *at al* found no significant correlation between quarantine and symptoms of PTSD, stress, anxiety, or depression in 26 Chinese residents [55]. Sleep disturbance (4.4%, 71 of 1602), and suicidal ideation (1.9%, 30 of 1602) have been detailed following quarantine and research concerning emergency department attendances found that 11.31% (25 of 221) involved severe alcohol intoxication in the three weeks after lockdown compared to 0.88% (1 of 117) in the preceding period [29,52].

Social repercussions, including stigma and behavioural modifications, have been investigated in HCWs [51]. Duy *et al* reported 34.43% (21 of 61) expressed guilt for isolating, 18.03% (11 of 61) felt unsafe in their profession, and many described discriminatory experiences [51]. However, only 1.64% (1 of 61) detailed undertaking efforts to avoid COVID-19 related work after quarantine [51].

### Medium-term consequences of lockdown measures (1-6 months)

Symptoms of depression, anxiety, and trauma and stressor related disorders have been identified as medium-term after-effects following lockdown [17,22-24,26,27,45,48]. Behavioural and emotional repercussions, social exclusion, stigmatisation, changes to inpatient biochemical markers, weight, and dietary behaviours have also been established to occur one to six months after lockdown measures ended [17,33,34,36-38,40-42].

#### Severe acute respiratory syndrome

Three studies investigated medium-term PTSD symptoms following the SARS outbreak [17,26,45]. Hawryluck *et al* and Reynolds *et al* found that following Toronto-based home or workplace quarantine the prevalence of significant PTSD symptomatology was 28∙9% (35 of 129) and 14∙6% (148 of 1057) respectively [17,26]. Chong *et al*, however, found no correlation between quarantine and PTSD or psychiatric morbidity in Taiwanese HCWs [45]. Symptoms of depression and suicidal ideation were associated with quarantine [26,56]. Hawryluck *et al* described significant depression symptoms in 31∙2% of their cohort [26]. Although precise time-frames are unclear, one case-note review described increased suicide rates in those aged over 65 during and in the months after the SARS outbreak in Hong Kong with measures including reduced interpersonal contact, disruptions to social support, and experiences of disconnection highlighted as contributing factors [56]. Identified risk factors for symptoms of PTSD and depression included quarantine periods longer than ten days and lower household income [26]. Despite governmental compensation, Reynolds *et al* associated quarantine with household income reduction [17].

Behavioural changes and emotional reactions were observed [17,37,40]. Reynolds *et al* found that 53∙7% (568 of 1057) avoided those sneezing or coughing, whilst 25∙7% (272 of 1057) refrained from entering crowded public spaces up to four months after lockdown [17]. Individuals continued performing behaviours, including frequent hand washing, and several participants reported extending their quarantine or limiting social contact beyond public guidance [17,40]. A study involving nurses associated avoidance coping behaviours, including missing work and avoiding patient contact, with home or workplace quarantine [37]. Increased anger correlated with longer lengths of quarantine, although emotional exhaustion was not associated [37].

Stigmatisation following quarantine was reported in four studies [17,36,40,41]. Experiences ranged from heightened attention to social ostracization in settings including day-care facilities, workplaces, and private social gatherings, leading some HCWs to deny their occupation or conceal they had quarantined [17,40,41]. However, perceived discrimination reduced to 4∙0% (36 of 903) from 48∙7% (440 of 903) for residents in Hong Kong three months after lockdown measures ended compared to immediately after quarantine [36].

#### Middle East respiratory syndrome

Symptoms of anxiety, requirements for psychiatric support, and changes to biochemical parameters are identified medium-term consequences following the MERS outbreak in South Korea [27,33,38,57].

A study quantifying mental health service utilisation by those quarantined demonstrated that 19∙6% (1221 of 6231) experienced psychological and emotional difficulties during quarantine, with 28∙7% (350 of 1221) of these requiring follow-up after measures ended [57]. Jeong *et al* described 7∙6% (126 of 1656) of residents reporting anxiety symptoms during isolation, decreasing to 3∙0% (50 of 1656) four to six months later, similar to general population prevalence [27]. Factors associated with increased anxiety symptoms included insufficient supply provision, lacking access to social networking facilities, psychiatric comorbidities, and financial concerns [27].

Two studies investigated biological parameters in haemodialysis patients during and after quarantine or isolation [33,38]. One study reported impaired haemodialysis efficacy and albumin levels during isolation which significantly improved three months later [38]. Another study reported normal haemodialysis efficacy, however, detected continuously elevated stress markers in patients compared to HCWs quarantined alongside them [33].

#### Coronavirus disease 2019

Repercussions for psychological well-being, dietary habits, and weight have been identified medium-term outcomes following COVID-19 lockdown measures [22-24,42].

Psychological distress, comprising symptoms of depression and anxiety disorders, was found to increase during measures for American participants with or without mental health diagnoses [22]. Although levels of distress returned to baseline in both cohorts after measures ended, initial distress severity was significantly higher in those with pre-existing psychiatric diagnoses [22]. Symptoms of anxiety, PTSD, and depression were identified during lockdown in the general public and HCWs by Lu *et al*, with HCW PTSD (16.2%, 62 of 382) and any depression (57.9%, 221 of 382) symptomatology significantly higher compared to general population prevalence, (11.7%, 121 of 1035) and (45.7%, 473 of 1035) respectively [24]. Similarly, overall symptom predominance decreased following measures lifting [24]. Conversely, symptoms of stress, anxiety, and depression in a study of Chinese students decreased during national lockdown, and subsequently increased after measures were removed [23]. Minimal changes to psychological well-being were described by Zhou *et al* six weeks after national lockdown ended in China [58].

Two studies described consequences to dietary behaviours and weight [34,42]. Over half of respondents in one study (n = 1994) reduced their consumption of seafood, raw food, and imported frozen food whilst increasing the frequency of home-cooking after lockdown in China [34]. Several participants also continued engaging in unhealthy dietary behaviours including drinking vinegar (9.8%, 195 of 1994) to prevent infection [34]. Zarah *et al* found that although most American respondents did not report dietary changes following lockdown, 38% (1191 of 3133) described weight gain, and increased consumption of sweets and salty snacks were nevertheless noted [42].

### Long-term consequences of lockdown measures (>6 months)

Psychological distress, alcohol abuse/dependence and financial concerns have been described to persist over six months after lockdown measures end [16,25,31,32,43,54]. Several at-risk groups for long-term psychological morbidity were identified [16,25,31,32,43,54].

#### Severe acute respiratory syndrome

Three studies analysed survey responses from HCWs, 18∙8% (103 of 549) of whom were quarantined, three years following the SARS outbreak in Beijing [31,32,43]. Quarantine was significantly associated with increased alcohol abuse/dependence, PTSD, and depression symptoms [31,32,43]. Identified groups at-risk for exhibiting PTSD symptoms over six months after measures ended were HCWs who experienced prolonged contact with infectious patients or those who had close relations diagnosed with SARS [32]. Risk factors for depression symptoms included concurrent PTSD symptoms, work-related stressors, being single, and prior experiences of traumatic events [31]. Mihashi *et al* measured the prevalence of psychological disorders in printing company workers, university staff, and students during and after hospital isolation or home quarantine [54]. During these measures, 24∙6% (46 of 187) reported symptoms indicating psychological disorder increasing to 26∙2% (49 of 187) seven to eight months later, however, this did not achieve statistical significance [54]. Income reduction was identified as the principally associated risk factor [54].

#### Middle East respiratory syndrome

Significant long-term PTSD symptoms were identified in 17∙9% (12 of 67) of quarantined haemodialysis patients in South Korea, however, lengths of isolation of 16 days or more were associated with lower symptomatology [16]. Females were also at higher risk of exhibiting PTSD symptoms over six months after lockdown measures ended [16].

#### Ebola

Another study investigated PTSD, anxiety, and depression symptomatology in 3564 residents in Sierra Leone approximately 14 months after the start of the Ebola outbreak [25]. Knowing somebody who had been quarantined or having personal relations with suspicion or diagnosis of Ebola was independently associated with increased PTSD, anxiety, and depression symptoms [25]. Higher and ongoing perceived disease threat was also associated with symptom prevalence [25].

### Impacts of lockdown measures on primary caregivers and children

Social and psychological consequences in families and children were evident following SARS and H1N1-related quarantine [18,53]. A third of primary caregivers, surveyed approximately one to six months after quarantine or isolation, reported their children began utilising mental health services during or after measures [18]. Diagnoses including generalised anxiety disorder, acute stress disorder, adjustments disorder, grief, and PTSD [18]. 28% (27 of 98) of quarantined parents met diagnostic cut-offs for PTSD compared to 5∙8% (17 of 299) not quarantined, whilst 30% of children also met diagnostic criteria [18]. Significant associations between children experiencing containment measures, paediatric PTSD symptoms, and parents concurrently displaying PTSD symptoms were found [18]. A qualitative study of parents and children described children developing anxious behaviours including increased signs of distress when parents left for work two months after hospital isolation [53].

## DISCUSSION

### What have we learned from previous lockdown measures and what are the implications for current lockdown measures?

#### Principal findings

Lockdown measures are a venerable method to contain infectious disease outbreaks, however, profound biopsychosocial impacts, ranging from acute stress reactions to chronic psychopathology, are apparent [7]. Economic repercussions may be compounded by social obstacles in returning to work (**Table 2**) [26-28,39]. Certain groups, such as those lacking financial security, are therefore particularly vulnerable to continued financial hardship [22,26-28,30,39,50,54]. Enduring behavioural and social effects and adjustments to workplace practice can impede return to social and professional normalcy which may require extensive support including workplace modifications [15,17,28,37,39-41,51]. Acute alcohol intoxication, weight gain, and dietary changes have also been described [34,42,52]. Physical, emotional, and social well-being of HCWs during outbreaks may improve following self-quarantine compared to parameters observed during clinical work [44]. However, no differences in the degree of psychiatric symptoms in other workforces following COVID-19 measures were found [19]. National interventions to alleviate individually perceived disease threat and improve social support may reduce psychological morbidity [25,28,56]. Actively approaching vulnerable individuals and ensuring publicly accessible mental health services may reduce and prevent mental health burden [19,28]. However, comprehensive research analysing effective interventions to mitigate biopsychosocial aftereffects of lockdown is lacking.

**Table 2:** Suggested strategies to mitigate harmful biopsychosocial consequences of lockdown measures from included studies

Tailored interventions according to time-elapsed, including acute psychological support and longer-term management targeting maladaptive coping mechanisms [15,16,18,25,27,31,32,34,35,39,41,43,45,57]
Early identification and active follow-up of high risk and vulnerable groups [16-22,25-28,30-32,35,39,43,50,53,54]
Public health authorities to deliver clear and accurate information and educational resources [17,19,26-28,36,39-41,54,55]
Provision of financial aid, supplies, and hygiene-enhancing policies [17,19,25,27,30,39,40,54,55]
Employer attention to employees’ physical and psychological well-being [19,26,36,37,40,51]
Promotion of social connectivity, networking, and support [20,25,27,28,53,56]

#### Previous studies

Brooks *et al* reviewed psychological consequences of quarantine primarily focusing on interventions and outcomes during quarantine [4]. Acute stress reactions, PTSD, anxiety, and depression symptoms were identified [4]. Behavioural changes, reluctance to re-enter work, and deteriorating work performance were also described [4]. Financial loss consequent to quarantine was an evident stressor for psychological well-being [4]. Brooks *et al* highlighted the importance of social networking facilities in avoiding frustration, boredom and anxiety, and emphasised the importance of clear public health messages [4]. Recent evidence indicated that measures introduced in China, including city-wide lockdown, swift isolation of confirmed and suspected cases, effective contact tracing, and workplace adjustments likely helped improve public confidence [19,30]. Brooks *et al* additionally reported that stigmatisation following quarantine could be ameliorated through public education [4].

Previous reviews had not investigated suicidality, biological markers including biochemical parameters and weight change, dietary behaviours, or psychiatric symptoms in returning workforces after lockdown measures. The importance of social support, relationships, and their correlation with mental health well-being have also not been addressed. Social barriers compounding financial hardship, the implications of perceived disease risk, and the significance of childhood isolation from primary caregivers were similarly unexamined. This review exclusively explored repercussions once lockdown measures were lifted. To our knowledge, this is also the first review to report a possible restorative effect of quarantine in HCWs by improving emotional, physical, and social well-being.

### Implications

Individualised, accessible, and timely support delivered on multidimensional platforms focusing on short, medium, and long-term consequences may restrict psychological distress, economic repercussions, stigmatisation, and assist high-risk groups. Identified at-risk groups include those with pre-existing psychiatric diagnoses, children, elderly, HCWs, socially isolated individuals, and hospital inpatients. Short to medium-term interventions may require acute psychological support directed at trauma processing. Medium to long-term repercussions could necessitate approaches reforming maladaptive coping mechanisms, behaviours including substance misuse, and addressing psychological morbidity, particularly PTSD and depression. Prioritising early public education may prevent short to medium-term stigmatisation of quarantining individuals. Economic adversity can have profound short and long-term effects therefore financial support, modification of work environments, and encouraging employment could enable financial rehabilitation and prevent mental health sequela. Actively identifying and directing services towards vulnerable groups could similarly prevent psychological morbidity. Social connectedness, known to enhance health, may require tailored communication initiatives to promote re-integration to social and professional activities following lockdown measures [59]. Arranging sufficient social support and ensuring access to essential items, could additionally mitigate negative outcomes.

### Strengths and limitations

This review includes studies from a range of infectious outbreaks including SARS, MERS, Ebola, H1N1 and COVID-19. The present review incorporated a comprehensive search strategy, manual analysis of references lists, and inclusion of several languages. Multiple researchers employed independent study identification and data extraction reducing bias and increasing study yield.

Despite these strengths, most eligible studies were cross-sectional and mainly comprised research examining SARS and COVID-19 outbreaks. Diversity in disease-cause and consequently applied lockdown measures presented significant heterogeneity, occasionally involved small sample sizes, and investigated specific populations from diverse cultural backgrounds and professions which may yield results not widely applicable or comparable. Notably, at the time of writing the COVID-19 pandemic was ongoing with lockdown measures varying by country and region albeit with temporary easing and tightening according to fluctuating infection rates. Variations in outcomes and measurement tools may have produced unstandardised diagnostic symptom identification. Comparators were not always included by reviewed studies, and research on other lockdown measures including mandatory use of masks and school closures, were rare or unavailable. No randomised controlled trials were identified. Given the evolution, fluctuating lockdown measures, and unprecedented nature of the COVID-19 pandemic, the relevance of past interventions and outcomes are difficult to predict. While consequences of lockdown measures have been described, the biopsychosocial impact of not implementing such measures in a randomised trial is ethically challenging in a research environment hence leaving retrospective analysis as the most pragmatic option.

### Future research

Whilst there is strong evidence that the end of lockdown will be accompanied by harmful biopsychosocial effects, randomised controlled trials of preventive measures are lacking. Further research defining high risk groups and investigating tailored preventive approaches is needed. Further pandemics in addition to resurgences of COVID-19 are expected, therefore researching mitigating effects of future lockdowns is urgently required.

## CONCLUSIONS

COVID-19 lockdown measures are unprecedented and have highlighted the need to rapidly produce high-quality research evaluating the effectiveness of actions and identifying further consequences. Establishing research in the eventuality of future lockdown periods is crucial to producing an evidence base that can inform decisions and recommendations. Lockdown can be traumatic, provoke mental health deterioration, induce financial hardship, and repercussions for social and behavioural conduct may impede social connectedness and impact professional practice. Offering support tailored to short, medium, and long-term consequences may help overcome acute and persisting hardship and prevent psychological morbidity. Establishing relevant research informing prompt, coherent, and consistent public health guidance and social support could be essential in mitigating negative outcomes following lockdown measures.

## Additional material

Online Supplementary Document

## References

[1] Anderson RM Heesterbeek H Klinkenberg D Hollingsworth TD How will country-based mitigation measures influence the course of the COVID-19 epidemic? Lancet. 2020;395:931-4. 10.1016/S0140-6736(20)30567-5 32164834 PMC7158572

[2] World Health Organization. Considerations in adjusting public health and social measures in the context of COVID-19:Interim guidance. Available: https://www.who.int/publications/i/item/considerations-in-adjusting-public-health-and-social-measures-in-the-context-of-covid-19-interim-guidance. Accessed: 20 November 2020.

[3] Weible CM Nohrstedt D Cairney P Carter DP Deserai C Durnová A COVID-19 and the policy sciences: initial reactions and perspectives. Policy Sci. 2020. Online ahead of print. 10.1007/s11077-020-09381-4 32313308 PMC7165254

[4] Brooks SK Webster RK Smith LE Woodland L Wessely S Greenberg N The psychological impact of quarantine and how to reduce it: rapid review of the evidence. Lancet. 2020;395:912-20. 10.1016/S0140-6736(20)30460-8 32112714 PMC7158942

[5] Lippi G Henry BM Bovo C Sanchis-Gomar F Health risks and potential remedies during prolonged lockdowns for coronavirus disease 2019 (COVID-19). Diagnosis (Berl). 2020;7:85-90. 10.1515/dx-2020-0041 32267243

[6] CDC. Social Distancing. Available: https://www.cdc.gov/coronavirus/2019-ncov/prevent-getting-sick/social-distancing.html. Accessed: 20 November 2020.

[7] Bensimon CM Upshur REG Evidence and effectiveness in decisionmaking for quarantine. Am J Public Health. 2007;97:S44-8. 10.2105/AJPH.2005.077305 17413076 PMC1854977

[8] Nussbaumer-Streit B Mayr V Dobrescu AI Chapman A Persad E Klerings I Quarantine alone or in combination with other public health measures to control COVID-19: a rapid review. Cochrane Database Syst Rev. 2020;4:CD013574. 32267544 10.1002/14651858.CD013574 PMC7141753

[9] Holmes EA O’Connor RC Perry VH Tracey I Wessely S Arseneault L Multidisciplinary research priorities for the COVID-19 pandemic: a call for action for mental health science. Lancet Psychiatry. 2020;7:547-60. 10.1016/S2215-0366(20)30168-1 32304649 PMC7159850

[10] Chen P Mao L Nassis GP Harmer P Ainsworth BE Li F Wuhan coronavirus (2019-nCoV): The need to maintain regular physical activity while taking precautions. J Sport Health Sci. 2020;9:103-4. 10.1016/j.jshs.2020.02.001 32099716 PMC7031771

[11] Fernandes N Economic effects of coronavirus outbreak (COVID-19) on the world economy. SSRN. 2020. . 10.2139/ssrn.3557504

[12] Van Lancker W Parolin Z COVID-19, school closures, and child poverty: a social crisis in the making. Lancet Public Health. 2020;5:e243-4. 10.1016/S2468-2667(20)30084-0 32275858 PMC7141480

[13] Lee J Mental health effects of school closures during COVID-19. Lancet Child Adolesc Health. 2020;4:421. 10.1016/S2352-4642(20)30109-7 32302537 PMC7156240

[14] Bavli I Sutton B Galea S Harms of public health interventions against covid-19 must not be ignored. BMJ. 2020;371:m4074. 10.1136/bmj.m4074 33139247

[15] Bai Y Lin CC Lin CY Chen JY Chue CM Chou P Survey of stress reactions among health care workers involved with the SARS outbreak. Psychiatr Serv. 2004;55:1055-7. 10.1176/appi.ps.55.9.1055 15345768

[16] Cho AJ Lee HS Lee YK Jeon HJ Park HC Jeong DW Post-traumatic stress symptoms in hemodialysis patients with MERS-CoV exposure. Biopsychosoc Med. 2020;14:9. 10.1186/s13030-020-00181-z 32308734 PMC7156895

[17] Reynolds DL Garay JR Deamond SL Moran MK Gold W Styra R Understanding, compliance and psychological impact of the SARS quarantine experience. Epidemiol Infect. 2008;136:997-1007. 10.1017/S0950268807009156 17662167 PMC2870884

[18] Sprang G Silman M Posttraumatic stress disorder in parents and youth after health-related disasters. Disaster Med Public Health Prep. 2013;7:105-10. 10.1017/dmp.2013.22 24618142

[19] Tan W Hao F McIntyre RS Jian L Jiang X Zhang L Is Returning to Work during the COVID-19 Pandemic Stressful? A Study on Immediate Mental Health Status and Psychoneuroimmunity Prevention Measures of Chinese Workforce. Brain Behav Immun. 2020;87:84-92. 10.1016/j.bbi.2020.04.055 32335200 PMC7179503

[20] Probst T Budimir S Pieh C Depression in and after COVID-19 lockdown in Austria and the role of stress and loneliness in lockdown: A longitudinal study. J Affect Disord. 2020;277:962-3. 10.1016/j.jad.2020.09.047 33065839 PMC7487145

[21] Grover R Dua P Juneja S Chauhan L Khurana A “Depression, Anxiety and Stress” in a Cohort of Registered Practicing Ophthalmic Surgeons, Post Lockdown during COVID-19 Pandemic in India. Ophthalmic Epidemiol. 2020. Online ahead of print. 10.1080/09286586.2020.1846757 33185487

[22] Daly M Robinson E Psychological distress and adaptation to the COVID-19 crisis in the United States. J Psychiatr Res. 2020. Online ahead of print. 10.1016/j.jpsychires.2020.10.035 33138985 PMC7588823

[23] Li WW Yu H Miller DJ Yang F Rouen C Novelty Seeking and Mental Health in Chinese University Students Before, During, and After the COVID-19 Pandemic Lockdown: A Longitudinal Study. Front Psychol. 2020;11:600739. 10.3389/fpsyg.2020.600739 33343473 PMC7744699

[24] Lu P Li X Lu L Zhang Y The psychological states of people after Wuhan eased the lockdown. PLoS One. 2020;15:e0241173. 10.1371/journal.pone.0241173 33180783 PMC7660514

[25] Jalloh MF Li W Bunnell RE Ethier KA O’Leary A Hageman KM Impact of Ebola experiences and risk perceptions on mental health in Sierra Leone, July 2015. BMJ Glob Health. 2018;3:e000471. 10.1136/bmjgh-2017-000471 29607096 PMC5873549

[26] Hawryluck L Gold WL Robinson S Pogorski S Galea S Styra R SARS control and psychological effects of quarantine, Toronto, Canada. Emerg Infect Dis. 2004;10:1206-12. 10.3201/eid1007.030703 15324539 PMC3323345

[27] Jeong H Yim HW Song YJ Ki M Min JA Cho J Mental health status of people isolated due to Middle East Respiratory Syndrome. Epidemiol Health. 2016;38:e2016048. 10.4178/epih.e2016048 28196409 PMC5177805

[28] Ko CH Yen CF Yen JY Yang MJ Psychosocial impact among the public of the severe acute respiratory syndrome epidemic in Taiwan. Psychiatry Clin Neurosci. 2006;60:397-403. 10.1111/j.1440-1819.2006.01522.x 16884438

[29] Ritish D Dinakaran D Chander R Murugesan M Ibrahim FA Parthasarathy R Mental health concerns in quarantined international air passengers during COVID-19 pandemic – An experiential account. Asian J Psychiatr. 2020;53:102364. 10.1016/j.ajp.2020.102364 32877856 PMC7446664

[30] Lei L Huang X Zhang S Yang J Yang L Xu M Comparison of Prevalence and Associated Factors of Anxiety and Depression among People Affected by versus People Unaffected by Quarantine during the COVID-19 Epidemic in Southwestern China. Med Sci Monit. 2020;26:e924609. 10.12659/MSM.924609 32335579 PMC7199435

[31] Liu X Kakade M Fuller CJ Fan B Fang Y Kong J Depression after exposure to stressful events: Lessons learned from the severe acute respiratory syndrome epidemic. Compr Psychiatry. 2012;53:15-23. 10.1016/j.comppsych.2011.02.003 21489421 PMC3176950

[32] Wu P Fang Y Guan Z Fan B Kong J Yao Z The psychological impact of the SARS epidemic on hospital employees in China: Exposure, risk perception, and altruistic acceptance of risk. Can J Psychiatry. 2009;54:302-11. 10.1177/070674370905400504 19497162 PMC3780353

[33] Kim YG Moon H Kim SY Lee YH Jeong DW Kim K Inevitable isolation and the change of stress markers in hemodialysis patients during the 2015 MERS-CoV outbreak in Korea. Sci Rep. 2019;9:5676. 10.1038/s41598-019-41964-x 30952879 PMC6450937

[34] Zhang J Zhao A Ke Y Huo S Ma Y Zhang Y Dietary Behaviors in the Post-Lockdown Period and Its Effects on Dietary Diversity: The Second Stage of a Nutrition Survey in a Longitudinal Chinese Study in. Nutrients. 2020;12:3269. 10.3390/nu12113269 33114499 PMC7693097

[35] Lee SM Kang WS Cho AR Kim T Park JK Psychological impact of the 2015 MERS outbreak on hospital workers and quarantined hemodialysis patients. Compr Psychiatry. 2018;87:123-7. 10.1016/j.comppsych.2018.10.003 30343247 PMC7094631

[36] Lee S Chan LYY Chau AMY Kwok KPS Kleinman A The experience of SARS-related stigma at Amoy Gardens. Soc Sci Med. 2005;61:2038-46. 10.1016/j.socscimed.2005.04.010 15913861 PMC7116975

[37] Marjanovic Z Greenglass ER Coffey S The relevance of psychosocial variables and working conditions in predicting nurses’ coping strategies during the SARS crisis: An online questionnaire survey. Int J Nurs Stud. 2007;44:991-8. 10.1016/j.ijnurstu.2006.02.012 16618485 PMC7094220

[38] Park HC Lee SH Kim J Kim DH Cho A Jeon HJ Effect of isolation practice on the transmission of middle east respiratory syndrome coronavirus among hemodialysis patients: A 2-year prospective cohort study. Medicine (Baltimore). 2020;99:e18782. 10.1097/MD.0000000000018782 32011472 PMC7220504

[39] Cava MA Fay KE Beanlands HJ McCay EA Wignall R The experience of quarantine for individuals affected by SARS in Toronto. Public Health Nurs. 2005;22:398-406. 10.1111/j.0737-1209.2005.220504.x 16229732

[40] DiGiovanni C Conley J Chiu D Zaborski J Factors influencing compliance with quarantine in Toronto during the 2003 SARS outbreak. Biosecur Bioterror. 2004;2:265-72. 10.1089/bsp.2004.2.265 15650436

[41] Robertson E Hershenfield K Grace SL Stewart DE The psychosocial effects of being quarantined following exposure to SARS: A qualitative study of Toronto health care workers. Can J Psychiatry. 2004;49:403-7. 10.1177/070674370404900612 15283537

[42] Zarah A Bin, Enriquez-marulanda J, Andrade JM. Relationship between Dietary Habits, Food Attitudes and Food Security Status among Adults Living within The United States Three Months Post-Mandated Quarantine: A Cross-Sectional Study. Nutrients. 2020;12:3486. 10.3390/nu12113468 PMC7697798 33198215

[43] Wu P Liu X Fang Y Fan B Fuller CJ Guan Z Alcohol abuse/dependence symptoms among hospital employees exposed to a SARS outbreak. Alcohol Alcohol. 2008;43:706-12. 10.1093/alcalc/agn073 18790829 PMC2720767

[44] Chen NH Wang PC Hsieh MJ Huang CC Kao KC Chen YH Impact of Severe Acute Respiratory Syndrome Care on the General Health Status of Healthcare Workers in Taiwan. Infect Control Hosp Epidemiol. 2007;28:75-9. 10.1086/508824 17230391

[45] Chong MY Wang WC Hsieh WC Lee CY Chiu NM Yeh WC Psychological impact of severe acute respiratory syndrome on health workers in a tertiary hospital. Br J Psychiatry. 2004;185:127-33. 10.1192/bjp.185.2.127 15286063

[46] Moher D Liberati A Tetzlaff J Altman DG The PRISMA Group Preferred reporting items for systematic reviews and meta-analyses: The PRISMA statement. PLoS Med. 2009;6:e1000097. 10.1371/journal.pmed.1000097 19621072 PMC2707599

[47] Tong A Flemming K McInnes E Oliver S Craig J Enhancing transparency in reporting the synthesis of qualitative research: ENTREQ. BMC Med Res Methodol. 2012;12:181. 10.1186/1471-2288-12-181 23185978 PMC3552766

[48] American Psychiatric Association. Diagnostic and statistical manual of mental disorders (5th ed.). Arlington, VA: American Psychiatric Association; 2013.

[49] Hong QN, Pluye P, Fàbregues S, Bartlett G, Boardman F, Cargo M, et al. Mixed Methods Appraisal Tool (MMAT), version 2018. Registration of Copyright (#1148552), Canadian Intellectual Property Office, Industry Canada.

[50] Chandola T Kumari M Booker CL Benzeval M The mental health impact of COVID-19 and lockdown-related stressors among adults in the UK. Psychol Med. 2020. Online ahead of print. 10.1017/S0033291720005048 33280639 PMC7783135

[51] Do Duy C Nong VM Van AN Thu D Thu ND Quang TN COVID-19 related stigma and its association with mental health of health- care workers after quarantined in Vietnam. Psychiatry Clin Neurosci. 2020;74:566-8. 10.1111/pcn.13120 32779787 PMC7404653

[52] Grigoletto V Cognigni M Occhipinti AA Abbracciavento G Carrozzi M Barbi E Rebound of Severe Alcoholic Intoxications in Adolescents and Young Adults After COVID-19 Lockdown. J Adolesc Health. 2020;67:727-9. 10.1016/j.jadohealth.2020.08.017 32943287 PMC7490634

[53] Koller DF Nicholas DB Goldie RS Gearing R Selkirk EK Bowlby and Robertson revisited: The Impact of isolation on hospitalized children during SARS. J Dev Behav Pediatr. 2006;27:134-40. 10.1097/00004703-200604000-00010 16682879

[54] Mihashi M Otsubo Y Yinjuan X Nagatomi K Hoshiko M Ishitake T Predictive Factors of Psychological Disorder Development During Recovery Following SARS Outbreak. Health Psychol. 2009;28:91-100. 10.1037/a0013674 19210022

[55] Wang C Pan R Wan X Tan Y Xu L Ho CS Immediate psychological responses and associated factors during the initial stage of the 2019 coronavirus disease (COVID-19) epidemic among the general population in China. Int J Environ Res Public Health. 2020;17:1729. 10.3390/ijerph17051729 32155789 PMC7084952

[56] Yip PSF Cheung YT Chau PH Law YW The impact of epidemic outbreak: The case of severe acute respiratory syndrome (SARS) and suicide among older adults in Hong Kong. Crisis. 2010;31:86-92. 10.1027/0227-5910/a000015 20418214

[57] Yoon MK Kim SY Ko HS Lee MS System effectiveness of detection, brief intervention and refer to treatment for the people with post-traumatic emotional distress by MERS: A case report of community-based proactive intervention in South Korea. Int J Ment Health Syst. 2016;10:51. 10.1186/s13033-016-0083-5 27504141 PMC4976505

[58] Zhou T Nguyen TT Zhong J Liu JA COVID-19 descriptive study of life after lockdown in Wuhan, China. R Soc Open Sci. 2020;7:200705. 10.1098/rsos.200705 33047032 PMC7540789

[59] Saeri AK Cruwys T Barlow FK Stronge S Sibley CG Social connectedness improves public mental health: Investigating bidirectional relationships in the New Zealand attitudes and values survey. Aust N Z J Psychiatry. 2018;52:365-74. 10.1177/0004867417723990 28803484

