# Supplementary material for: Learning from previous lockdown measures and minimising harmful biopsychosocial consequences as they end: A systematic review
Source: J Glob Health. 2021 May 22;11:05008. doi: 10.7189/jogh.11.05008 (PMC8141332; doi:10.7189/jogh.11.05008)
Supplement: Online Supplementary Document [file jogh-11-05008-s001.pdf]

Supplementary File

Table S1: Search strategy

**PubMed, 12th January 2021**
Disease Outbreaks [mh]
AND
pandemic OR epidemic OR severe acute respiratory syndrome OR sars OR covid* OR coronavirus OR middle east respiratory syndrome OR mers OR influenza OR flu OR ebola OR h1n1
AND
lockdown OR isolat* OR social distanc* OR physical distanc* OR quarantin* OR workplace closure OR school closure OR Quarantine [mh] OR Social Isolation [mh]
AND
biopsychosocial OR mental health OR psych* OR ptsd OR post-traumatic stress disorder OR stress OR stigma* OR suicid* OR depress* OR anxi* OR addict* OR alcohol* OR abuse OR insomnia OR sleep OR biomarkers OR Mental Health [mh] OR Mental Disorders [mh] OR Behavior and Behavior Mechanisms [mh] OR Substance-Related Disorders [mh] OR Domestic Violence [mh] OR Biomarkers [mh] OR Vital Signs [mh]
**Filters**
Species: humans<br>Article type: case reports, clinical study, clinical trial, comparative study, controlled clinical trial, journal article, multicenter study, observational study, pragmatic clinical trial, randomized controlled trial, twin study
**Other databases searched using keywords**
Ovid MEDLINE, Embase, PsychInfo, Web of Science and Scopus

Table S2: Assessment of study quality using Mixed Methods Appraisal Tool 2018

Qualitative studies*<br>Author (year)	Is the qualitative approach appropriate to answer the research question?	Are the qualitative data collection methods adequate to address the research question?	Are the findings adequately derived from the data?	Is the interpretation of results sufficiently substantiated by data?	Is there coherence between qualitative data sources, collection, analysis and interpretation?	Overall quality rating
Cava et al (2005)	1	1	1	1	1	5
DiGiovanni et al (2004)	1	1	1	0	1	4
Koller et al (2006)	1	1	1	1	1	5
Robertson et al (2004)	1	0	0	1	0	2
Yip et al (2010)	1	1	1	1	1	5
**Quantitative non-randomised studies*<br>Author (year)**	**Are the participants representative of the target population?**	**Are measurements appropriate regarding both the outcome and intervention (or exposure)?**	**Are the complete outcome data?**	**Are the confounders accounted for in the design and analysis?**	**During the study period, is the intervention administered (or exposure occurred) as intended?**	**Overall quality rating**
Bai et al (2004)	1	0	1	0	1	3
Chen et al (2007)	1	1	0	0	1	3
Chong et al (2004)	0	0	1	0	1	2
Jalloh et al (2015)	1	0	1	0	1	3
Ko et al (2006)	0	0	1	0	1	2
Lei et al (2020)	0	1	1	1	1	4
Liu et al (2012)	0	0	1	1	1	3
Marjanovic et al (2007)	1	1	1	0	1	4
Park et al (2020)	1	1	0	0	1	3
Ping et al (2008)	0	0	1	1	1	3
Ping et al (2009)	0	0	1	1	1	3
Wang et al (2020)	0	1	1	0	1	3
**Quantitative descriptive studies*<br>Author (year)**	**Is the sampling strategy relevant to address the research question?**	**Is the sample representative of the target population?**	**Are the measurements appropriate?**	**Is the risk of nonresponse bias low?**	**Is the statistical analysis appropriate to answer the research question?**	**Overall quality rating**
Chandola et al (2020)	1	1	1	0	1	4
Cho et al (2020)	1	0	1	0	1	3
Daly & Robinson (2020)	1	1	1	1	1	5
Duy et al (2020)	1	0	1	1	1	4
Grigoletto et al (2020)	1	1	1	1	1	5
Grover et al (2020)	1	0	1	0	1	3

**Hawryluck et al (2004)**	1	0	1	0	1	3
**Jeong et al (2016)**	1	1	1	0	1	4
**Kim et al (2019)**	1	0	0	1	1	3
**Lee et al (2018)**	1	0	1	0	1	3
**Li et al (2020)**	1	0	1	0	1	3
**Lu et al (2020)**	1	0	1	1	1	4
**Mihashi et al (2009)**	1	0	0	1	1	3
**Probst et al (2020)**	1	0	1	0	1	3
**Reynolds et al (2007)**	1	1	1	1	1	5
**Ritish et al (2020)**	1	1	0	1	0	3
**Tan et al (2020)**	1	1	1	0	1	4
**Yoon et al (2016)**	1	1	1	1	1	5
**Zarah et al (2020)**	1	0	1	1	1	4
**Zhang et al (2020)**	1	0	1	0	1	3
**Zhou et al (2020)**	1	0	1	0	0	2
**Mixed methods studies* Author (year)**	**Is there an adequate rationale for using a mixed methods design to address the research question?**	**Are the different components of the study effectively integrated to answer the research question?**	**Are the outputs of the integration of qualitative and quantitative components adequately interpreted?**	**Are divergences and inconsistencies between quantitative and qualitative results adequately addressed?**	**Do the different components of the study adhere to the quality criteria of each tradition of the methods involved?**	**Overall quality rating**
**Lee et al (2005)**	1	0	1	1	0	3
**Sprang & Silman (2013)**	0	1	1	0	0	2
***Study designs determined by mixed methods appraisal tool between qualitative, quantitative randomised controlled trials, quantitative non-randomised, quantitative descriptive and mixed methods**						

Appendix S1: ENTREQ and PRISMA Checklists

The ENTREQ Checklist

Item	Guide and description	Reported on page #
1. Aim	State the research question the synthesis addresses	5
2. Synthesis methodology	Identify the synthesis methodology or theoretical framework which underpins the synthesis, and describe the rationale for choice of methodology (e.g. meta-ethnography, thematic synthesis, critical interpretive synthesis, grounded theory synthesis, realist synthesis, meta-aggregation, meta-study, framework synthesis)	5-6
3. Approach to searching	Indicate whether the search was pre-planned (comprehensive search strategies to seek all available studies) or iterative (to seek all available concepts until they theoretical saturation is achieved)	5 & study protocol
4. Inclusion criteria	Specify the inclusion/exclusion criteria (e.g. in terms of population, language, year limits, type of publication, study type)	5-6
5. Data sources	Describe the information sources used (e.g. electronic databases (MEDLINE, EMBASE, CINAHL, psycINFO), grey literature databases (digital thesis, policy reports), relevant organisational websites, experts, information specialists, generic web searches (Google Scholar) hand searching, reference lists) and when the searches conducted; provide the rationale for using the data sources	5
6. Electronic Search strategy	Describe the literature search (e.g. provide electronic search strategies with population terms, clinical or health topic terms, experiential or social phenomena related terms, filters for qualitative research, and search limits)	5-6 & appendix 1
7. Study screening methods	Describe the process of study screening and sifting (e.g. title, abstract and full text review, number of independent reviewers who screened studies)	5-6
8. Study characteristics	Present the characteristics of the included studies (e.g. year of publication, country, population, number of participants, data collection, methodology, analysis, research questions)	6-7 & Table 1
9. Study selection results	Identify the number of studies screened and provide reasons for study exclusion (e.g. for comprehensive searching, provide numbers of studies screened and reasons for exclusion indicated in a figure/flowchart; for iterative searching describe reasons for study exclusion and inclusion based on modifications to the research question and/or contribution to theory development)	6-7 & Figure 1
10. Rationale for appraisal	Describe the rationale and approach used to appraise the included studies or selected findings (e.g. assessment of conduct (validity and robustness), assessment of reporting (transparency), assessment of content and utility of the findings)	6
11. Appraisal items	State the tools, frameworks and criteria used to appraise the studies or selected findings (e.g. Existing tools: CASP, QARI, COREQ, Mays and Pope [25]; reviewer developed	5-6

	tools; describe the domains assessed: research team, study design, data analysis and interpretations, reporting)	
12. Appraisal process	Indicate whether the appraisal was conducted independently by more than one reviewer and if consensus was required	5-6
13. Appraisal results	Present results of the quality assessment and indicate which articles, if any, were weighted/excluded based on the assessment and give the rationale	6 & appendix 2
14. Data extraction	Indicate which sections of the primary studies were analysed and how were the data extracted from the primary studies? (e.g. all text under the headings "results /conclusions" were extracted electronically and entered into a computer software)	6
15. Software	State the computer software used, if any	6
16. Number of reviewers	Identify who was involved in coding and analysis	5-6
17. Coding	Describe the process for coding of data (e.g. line by line coding to search for concepts)	6
18. Study comparison	Describe how were comparisons made within and across studies (e.g. subsequent studies were coded into pre-existing concepts, and new concepts were created when deemed necessary)	6
19. Derivation of themes	Explain whether the process of deriving the themes or constructs was inductive or deductive	6
20. Quotations	Provide quotations from the primary studies to illustrate themes/constructs, and identify whether the quotations were participant quotations of the author's interpretation	n/a
21. Synthesis output	Present rich, compelling and useful results that go beyond a summary of the primary studies (e.g. new interpretation, models of evidence, conceptual models, analytical framework, development of a new theory or construct)	7-11

PRISMA 2009 Checklist

Section/topic	#	Checklist item	Reported on page #
**TITLE**			
Title	1	Identify the report as a systematic review, meta-analysis, or both.	1
**ABSTRACT**			
Structured summary	2	Provide a structured summary including, as applicable: background; objectives; data sources; study eligibility criteria, participants, and interventions; study appraisal and synthesis methods; results; limitations; conclusions and implications of key findings; systematic review registration number.	3
**INTRODUCTION**			
Rationale	3	Describe the rationale for the review in the context of what is already known.	5
Objectives	4	Provide an explicit statement of questions being addressed with reference to participants, interventions, comparisons, outcomes, and study design (PICOS).	5
**METHODS**			
Protocol and registration	5	Indicate if a review protocol exists, if and where it can be accessed (e.g., Web address), and, if available, provide registration information including registration number.	5
Eligibility criteria	6	Specify study characteristics (e.g., PICOS, length of follow-up) and report characteristics (e.g., years considered, language, publication status) used as criteria for eligibility, giving rationale.	5-6
Information sources	7	Describe all information sources (e.g., databases with dates of coverage, contact with study authors to identify additional studies) in the search and date last searched.	5
Search	8	Present full electronic search strategy for at least one database, including any limits used, such that it could be repeated.	Appendix 1
Study selection	9	State the process for selecting studies (i.e., screening, eligibility, included in systematic review, and, if applicable, included in the meta-analysis).	5-6
Data collection process	10	Describe method of data extraction from reports (e.g., piloted forms, independently, in duplicate) and any processes for obtaining and confirming data from investigators.	6
Data items	11	List and define all variables for which data were sought (e.g., PICOS, funding sources) and any assumptions and simplifications made.	5-6

Risk of bias in individual studies	12	Describe methods used for assessing risk of bias of individual studies (including specification of whether this was done at the study or outcome level), and how this information is to be used in any data synthesis.	6
Summary measures	13	State the principal summary measures (e.g., risk ratio, difference in means).	6
Synthesis of results	14	Describe the methods of handling data and combining results of studies, if done, including measures of consistency (e.g., I^2) for each meta-analysis.	6

Section/topic	#	Checklist item	Reported on page #
Risk of bias across studies	15	Specify any assessment of risk of bias that may affect the cumulative evidence (e.g., publication bias, selective reporting within studies).	6
Additional analyses	16	Describe methods of additional analyses (e.g., sensitivity or subgroup analyses, meta-regression), if done, indicating which were pre-specified.	6
**RESULTS**			
Study selection	17	Give numbers of studies screened, assessed for eligibility, and included in the review, with reasons for exclusions at each stage, ideally with a flow diagram.	Figure 1
Study characteristics	18	For each study, present characteristics for which data were extracted (e.g., study size, PICOS, follow-up period) and provide the citations.	Table 1
Risk of bias within studies	19	Present data on risk of bias of each study and, if available, any outcome level assessment (see item 12).	7 & appendix 2
Results of individual studies	20	For all outcomes considered (benefits or harms), present, for each study: (a) simple summary data for each intervention group (b) effect estimates and confidence intervals, ideally with a forest plot.	7-11 & figure 2
Synthesis of results	21	Present the main results of the review. If meta-analyses are done, include for each, confidence intervals and measures of consistency.	7-11
Risk of bias across studies	22	Present results of any assessment of risk of bias across studies (see Item 15).	7 & appendix 2
Additional analysis	23	Give results of additional analyses, if done (e.g., sensitivity or subgroup analyses, meta-regression [see Item 16]).	7-11

DISCUSSION			
Summary of evidence	24	Summarize the main findings including the strength of evidence for each main outcome; consider their relevance to key groups (e.g., healthcare providers, users, and policy makers).	11-13
Limitations	25	Discuss limitations at study and outcome level (e.g., risk of bias), and at review-level (e.g., incomplete retrieval of identified research, reporting bias).	12
Conclusions	26	Provide a general interpretation of the results in the context of other evidence, and implications for future research.	13
**FUNDING**			
Funding	27	Describe sources of funding for the systematic review and other support (e.g., supply of data); role of funders for the systematic review.	6

From: Moher D, Liberati A, Tetzlaff J, Altman DG, The PRISMA Group (2009). Preferred Reporting Items for Systematic Reviews and Meta-Analyses: The PRISMA Statement. PLoS Med 6(7): e1000097. doi:10.1371/journal.pmed1000097

For more information, visit: **www.prisma-statement.org**.
